# Role of Leptin in the Activation of Immune Cells

**DOI:** 10.1155/2010/568343

**Published:** 2010-03-23

**Authors:** Patricia Fernández-Riejos, Souad Najib, Jose Santos-Alvarez, Consuelo Martín-Romero, Antonio Pérez-Pérez, Carmen González-Yanes, Víctor Sánchez-Margalet

**Affiliations:** Department of Clinical Biochemistry, Virgen Macarena University Hospital, University of Seville, Av. Dr. Fedriani 3, 41071 Seville, Spain

## Abstract

Adipose tissue is an active endocrine organ that secretes various humoral factors (adipokines), and its shift to production of proinflammatory cytokines in obesity likely contributes to the low-level systemic inflammation that may be present in metabolic syndrome-associated chronic pathologies such as atherosclerosis. Leptin is one of the most important hormones secreted by adipocytes, with a variety of physiological roles related to the control of metabolism and energy homeostasis. One of these functions is the connection between nutritional status and immune competence. The adipocyte-derived hormone leptin has been shown to regulate the immune response, innate and adaptive response, both in normal and pathological conditions. The role of leptin in regulating immune response has been assessed in vitro as well as in clinical studies. It has been shown that conditions of reduced leptin production are associated with increased infection susceptibility. Conversely, immune-mediated disorders such as autoimmune diseases are associated with increased secretion of leptin and production of proinflammatory pathogenic cytokines. Thus, leptin is a mediator of the inflammatory response.

## 1. Introduction

White adipose tissue plays a very important role in the energetic balance of mammals. This tissue is specialized in storing lipids and supplying fuel to the whole body whenever it is necessary. In order to face energetic requirements, adipocytes regulate fatty acid mobilization in response to catabolic and anabolic stimuli. However, adipose tissue is not only a reserve organ; it is also an endocrine organ able to release hormones, peptides, and cytokines (adipokines) that affect both the energetic status and the immune system [1]. Leptin is one of the most important hormones secreted by adipose tissue [2] and its implication in energetic homeostasis at central level has been largely described [3].

Rather than a “fasting signal”, leptin is a signal of starvation, in that a falling serum leptin concentration leads to neurohumoral and behavioural changes, trying to preserve energy reserves for vital functions. Thus, during fasting period and after reduction of body fat mass, there is a decrease in leptin levels that leads to a reduction in total energy expenditure to provide enough energy for the function of vital organs, that is, the brain, the heart, and the liver [4]. Even though these effects of leptin decrease are aimed to improve the survival chances under starving conditions, the fall in leptin levels may lead to immune suppression [5], in addition to other neuroendocrine alterations affecting adrenal, thyroid, and sexual/reproductive function [6]. At least, these alterations observed during fasting parallel the decrease in circulating leptin levels. In fact, both ob/ob mice (lacking leptin secretion) and db/db mice (lacking leptin receptor) are not only obese but they also show the immune/endocrine deficiencies observed during starvation [5, 7]. Moreover, it has been recently shown that leptin withdrawal during 8 days in experimental animals leads to the same effects regarding central control of endocrine systems including sexual function [8]. Even in humans, it has been found that leptin levels are associated with immune response in malnourished infants, which have low plasma leptin and impaired immune response [9]. Moreover, leptin signaling deficiency impairs humoral and cellular immune responses. The leptin receptor Ob-Rb is expressed by B and T lymphocytes, suggesting that leptin regulates directly the B and T cell responses [10].

The leptin modulation of the immune system is also mediated by the regulation of hematopoiesis and lymphopoiesis [7, 11] Thus, seven days of provision of recombinant leptin promoted substantial lymphopoiesis, with a twofold increase of the numbers of B cells in the marrow of obese mice while doubling and tripling, respectively, the numbers of pre-B and immature B cells. Twelve days of supplementation brought these subpopulations to near-normal proportions. Leptin treatment also facilitated myelopoiesis such that the marrow of the obese mice contained normal numbers of monocytes and granulocytes after 7 days [12].

**Figure 1:**
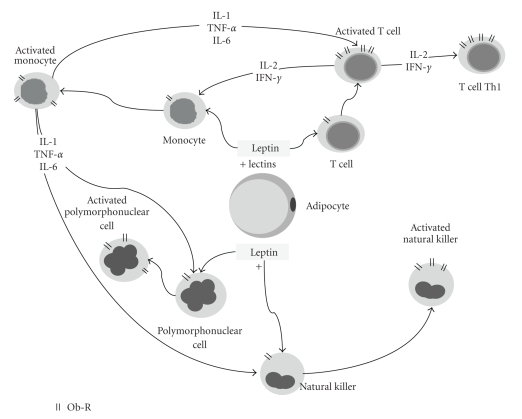
Role of leptin activating immune-competent cells.

Modulation of the immune system by leptin is exerted at the development, proliferation,antiapoptotic,maturation, and activation levels [13]. In fact, leptin receptors have been found in neutrophils, monocytes, and lymphocytes, and the leptin receptor belongs to the family of class I cytokine receptors. Moreover, leptin activates similar signaling pathways to those engaged by other members of the family [14]. The overall leptin action in the immune system is a proinflammatory effect, activating proinflammatory cells, promoting T-helper 1 responses, and mediating the production of the other proinflammatory cytokines, such as tumor necrosis factor-*α*, interleukin (IL)-2, or IL-6. Leptin receptor is also upregulated by proinflammatory signals. In this paper, we will summarize data from literature that demonstrate the positive regulation of the immune response by leptin (Figure 1) [14].

## 2. Leptin Activation of Innate Immunity

The primary amino acid sequence of leptin indicated that it could belong to the long-chain helical cytokine family [15], such as IL-2, IL-12, and GH. In fact, leptin receptor (Ob-R) shows sequence homology to members of class I cytokine receptor (gp130) superfamily [16] that includes the receptor for IL-6, leucocyte inhibitory factor (LIF), and granulocyte colony-stimulating factor (G-CSF). Moreover, Ob-R has been shown to have the signaling capabilities of IL-6-type cytokine receptors [16], activating JAK-STAT, PI3K, and MAPK signaling pathways [14]. In this context, a role for leptin in the regulation of innate immunity has been proposed [14, 17].

Consistent with this role of leptin in the mechanisms of immune response and host defense, circulating leptin levels are increased upon infectious and inflammatory stimuli such as LPS, turpentine, and cytokines [18, 19]. On the other hand, unlike other members of the IL-6 family, it is not clear that leptin may induce the expression of acute phase proteins, and contradictory data have been provided [19, 20]. The role of leptin regulating innate immunity has been previously reviewed [6]. 

### 2.1. Leptin Activation of Monocytes/Macrophages

Studies of rodents with genetic abnormalities in leptin or leptin receptors revealed obesity-related deficits in macrophage phagocytosis and the expression of proinflammatory cytokines both in vivo and in vitro, whereas exogenous leptin upregulated both phagocytosis and the production of cytokines [21]. Besides, phenotypic abnormalities in macrophages from leptin-deficient, obese mice have been found [22]. More importantly, leptin deficiency increases susceptibility to infectious and inflammatory stimuli and is associated with dysregulation of cytokine production [19]. More specifically, murine leptin deficiency alters Kupffer cell production of cytokines that regulate the innate immune system. In this context, leptin levels increase acutely during infection and inflammation, and may represent a protective component of the host response to inflammation [23].

Human leptin was found to stimulate proliferation and activation of human circulating monocytes in vitro, promoting the expression of activation markers: CD69, CD25, CD38, and CD71, in addition to increasing the expression of monocytes surface markers, such as HLA-DR, CD11b, and CD11c [24]. Besides, leptin potentiates the stimulatory effect of LPS or PMA on the proliferation and activation of human monocytes. Moreover, leptin dose-dependently stimulates the production of proinflammatory cytokines by monocytes, that is, TNF-*α* and IL-6 [25] and enhances CC-chemokine ligand expression in cultured murine macrophage, through activation of a JAK2-STAT3 pathway [25]. The presence of both isoforms of the leptin receptor was also assessed. Later, it was found that leptin directly induces the secretion of interleukin 1 receptor antagonist in human monocytes [26] and upregulates IP-10 (interferon-gamma-inducible protein) in monocytic cells [27]. Moreover, in human monocytes it has been shown that leptin increased both statin-inhibitable free radical and cholesterol productions in vitro [28]. In addition, it accelerates cholesteryl ester accumulation in human monocyte-derived macrophages by increasing ACAT-1 expression via JAK2 and PI3K, thereby suppressing cholesterol efflux [29]. In alveolar macrophages leptin augments leukotriene synthesis [30].

A possible role of leptin as a trophic factor to prevent apoptosis has also been found in serum-depleted human monocytes [31], which is further supporting the role of leptin as a growth factor for the monocyte. Moreover, leptin regulates monocyte function as assessed by in vitro experiments measuring free radical production. Thus, leptin was shown to stimulate the oxidative burst in control monocytes [32], and binding of leptin at the macrophage cell surface increases lipoprotein lipase expression through oxidative stress- and PKC-dependent pathways. In this line, leptin has been found to increase oxidative stress in macrophages [33]. Finally, leptin could act as a monocyte/macrophage chemoattractant inducing in vitro maximal chemotactic responses at 1 ng/mL [34], mediating the inflammatory infiltrate [35], and inducing tissue factor expression in human peripheral blood mononuclear cells [36]. On the other hand, human leptin seems to downregulate oxidative burst in previously activated monocytes [32].

Dendritic cells belong more to the same cell lineage than to monocytes/macrophages and also present leptin receptors (OBRb) on the cell surface [37]. Thus, leptin has also been found to increase the production of IL-8, IL-12, IL-6, and TNF-*α*, whereas it decreases MIP-1-*α* production by dendritic cells. Similar to leptin effect on monocytes,it may increase the survival of dendritic cells, and it may also increase the expression of surface molecules, such as CD1a, CD80, CD83, or CD86. Leptin induces functional and morphological changes in human dendritic cells (DCs), directing them towards Th1 priming and promoting DC survival via the PI3K-Akt signaling pathway [38]. The involvement of leptin signaling in DCs survival and maturation has been observed in leptin receptor- (Ob-R-) deficient db/db mice. Db/db mice displayed markedly reduced expression of costimulatory molecules and a Th2-type cytokine profile, with poor capacity to stimulate allogenic T cell proliferation. Consistent with their impaired DCs phenotype and function, db/db DCs showed significantly downregulated activities of the PI3K/Akt pathway as well as STAT-3 and IkappaB-alpha. Moreover, the reduced DCs yielded in db/db bone marrow culture was attributed to significantly increased apoptosis, which was associated with dysregulated expression of Bcl-2 family genes [39].

The expression of leptin and leptin receptors has been demonstrated on mast cells, suggesting paracrine and/or autocrine immunomodulatory effects of leptin on mast cells [40].

### 2.2. Leptin Activation of Neutrophils

Human polymorphonuclear neutrophils (PMN) have been found to express leptin receptor in vitro and in vivo [41, 42]. However, Zarkesh-Esfahani et al. [43] demonstrated that neutrophils only express the short form of the leptin receptor, which is enough to signal inside the cell, enhancing the expression of CD11b and preventing apoptosis [42, 43]. Leptin delayed the cleavage of Bid and Bax, the mitochondrial release of cytochrome c and second mitochondria-derived activator of caspase, as well as the activation of both caspase-8 and caspase-3 in these cells [42]. Therefore, leptin seems to behave as a survival cytokine for PMN, similar to G-CSF.

Leptin promotes neutrophils chemotaxis [19, 44]. In fact, the chemoattractant effect is comparable to that of well-known formyl-methionyl-leucyl-phenylalanine (FMLP). Otherwise, when leptin acts as a uremic toxin it interferes with neutrophil chemotaxis [45] and inhibits neutrophil migration in response to classical neutrophilic chemoattractants and leptin is endowed with chemotactic activity toward neutrophils. The two activities, inhibition of the cell response to chemokines and stimulation of neutrophil migration, could be detected at similar concentrations. On the contrary, neutrophils exposed to leptin did not display detectable [Ca2+]i mobilization, oxidant production, or beta2-integrin upregulation [46].

Moreover, leptin also has a stimulating effect on intracellular hydrogen peroxide production in PMN although this effect seems to be mediated by the activation of monocytes [42]. More specifically, leptin modulates neutrophil phagocytosis of *Klebsiella pneumoniae* [47] and in diabetic patients' neutrophils, an increase in leptin serum levels has been correlated with the degree of CD11b expression [48].

On eosinophils, leptin could upregulate cell surface expression of adhesion molecules ICAM-1 and CD18 but suppress ICAM-3 and L-selectin. Moreover, leptin could also stimulate the chemokinesis of eosinophils and induce the release of inflammatory cytokines IL-1beta and IL-6 and chemokines IL-8, growth-related oncogene-alpha, and MCP-1 [49].

### 2.3. Natural Killer (NK) Cells

Human NK cells constitutively express both long and short forms of Ob receptor. Moreover, the leptin receptors can signal in NK cells, since leptin activates STAT3 phosphorylation in NK cells. Moreover, leptin increases IL-2 and perforin gene expression at the transcription levels in NK cells. Consistent with this role of leptin regulating NK cells, db/db mice have been found to have impaired NK cell function [50, 51].

Leptin actions in NK cells include cell maturation, differentiation, activation, and cytotoxicity [20]. Leptin enhances both the development and the activation of NK cells [50], increasing IL-12 and reducing the expression of IL-15 [51]. Besides, leptin mediates the activation of NK cells indirectly by modulation of IL-1*β*, IL-6, and TNF-*α* by monocytes and macrophages [36].

## 3. Leptin Modulation of Adaptive Immune Response

The role of leptin in cell-mediated immunity has been obtained working with ob/ob mice [18]. These mice have a decreased sensitivity of T cells to activating stimuli. Besides, these animals show atrophy of lymphoid organs [5–7], with a decrease in the number of circulating T cells and an increase in the number of monocytes. Besides, ob/ob mice have a decrease in the number of TNKCD4+ in the liver [52]. The ability of leptin preventing thymic atrophia is due to a direct antiapoptotic effect on T cells [7]. Thus, leptin treatment increases thymic expression of interleukin-7, an important soluble thymocyte growth factor produced by medullary thymic epithelial cells (TECs). The hormone leptin has an intrathymic role in maintaining healthy thymic epithelium and promoting thymopoiesis, which is revealed when thymus homeostasis is perturbed by endotoxemia. In this case, leptin treatment decreases in vivo apoptosis of double positive thymocytes and promotes proliferation of double negative thymocytes [53].

In the thymus, Fyn acts as a tyrosine kinase that transduces the leptin signal independently of JAK2 activation and mediates some of the immunomodulatory effects of leptin in this tissue. The tyrosine kinase, Fyn, is constitutively associated with the Ob-R in thymic cells. Following a leptin stimulus, Fyn undergoes an activating tyrosine phosphorylation and a transient association with IRS1 [54].

Acute deficiency of leptin has a potent effect on the immune system, which is even higher than that observed in ob/ob mice (genetic defect). Acute hypoleptinemic mice show a higher decrease in the total number of thymocytes, and double number of apoptotic cells than ob/ob mice. Moreover, the acute deficiency of leptin also causes a decrease in splenic cellularity, which does not occur in ob/ob mice, even though they have a smaller spleen than control mice [8]. Both ob/ob and db/db mice show defects in cell-mediated immune response which lead to impaired reaction of delayed hypersensibility, suppression of skin allograft rejection, and inhibition of footpad swelling by recall antigens [6, 55–57]. In recent studies, leptin enhanced in vivo lymphocyte proliferation in Siberian hamsters (*Phodopus sungorus*) and increased splenocyte proliferation in mice [58] as well as it increased percentage of T cells, particularly CD4+ Th cells, in peritoneal fluid of patients with endometriosis [59].

Lord et al. 1998 [5], demonstrated that mouse lymphocytes express the long form of leptin receptor, and that leptin modulates in these cells cytokine production. Besides, leptin also regulates the number and activation of T lymphocytes. The proliferative response to leptin in mice seems to be produced in naive T cells (CD4+CD45RA+), whereas it has been shown that leptin inhibits proliferation of memory T cells (CD4+CD45RO+) [5]. Leptin provides a survival signal in double positive T cells (CD4+CD8+) and simple positive CD4+CD8− thymocytes during thymic maturation [7]. In addition, leptin promotes the expression of adhesion molecules in CD4+ T cells, such as VLA-2 (CD49b) or ICAM-1 (CD54) [5, 18].

More recently, we have reviewed the role of human leptin on T cell response [14]. Human leptin alone is not able to activate human peripheral blood lymphocytes in vitro [23] even though leptin receptor is present and activated in T lymphocytes upon leptin stimulation, fully triggering the intracellular signal transduction, as we have also assessed [60, 61]. However, when T lymphocytes are costimulated with PHA or concanavalin A (Con A), leptin dose-dependently enhances the proliferation and activation of cultured T lymphocytes, achieving maximal effect at 10 nM concentration [62]. Thus, leptin increases the expression of early activation markers such as CD69, as well as the expression of late activation markers, such as CD25, or CD71 in both CD4+ and CD8+ T lymphocytes in the presence of suboptimal concentrations of activators such as PHA (2 *μ*g/mL) and after stimulation with PMA-ionomycin [63]. However, when maximal concentrations of PHA or Con A are employed, leptin has no further effect. These effects of leptin on T lymphocytes are observed even in the absence of monocytes, suggesting a direct effect of human leptin on circulating T lymphocytes when they are costimulated [62]. The activation of T cells induces the expression of the long isoform of the Ob receptor [63]. The need for costimulation with mitogens to get the effect of leptin in lymphocytes may be partly explained by this effect of activation, increasing leptin receptor expression in T lymphocytes. Besides, these data suggest that the leptin receptor may be regulated in a similar way to other cytokine receptors, such as the IL-2 receptor (CD25).

Human leptin not only modulates the activation and proliferation of human T lymphocytes but also enhances cytokine production induced by submaximal concentrations of PHA [62]. Thus, human leptin enhances the production of IL-2 and IFN-*γ* in stimulated T lymphocytes. It had been previously shown in mice that leptin can enhance cognate T cell response, skewing cytokine responses towards a Th1 phenotype in mice [5]. These data are in agreement with the observation of the leptin effect on anti-CD3 stimulation of T cells, which increases the production of the proinflammatory cytokine IFN-*γ* [64]. The effect of leptin polarizing T cells towards a Th1 response seems to be mediated by stimulating the synthesis of IL-2, IL-12, and IFN-gamma and the inhibition of the production of IL-10 and IL-4 [36, 62]. Th1 polarization has been correlated with hyperleptinemia in hemodialysis patients [65] and the protection of ob/ob mice in Th1- as well as in Th2-dependent inflammation is provided by a decreased expression of the key transcription factors for Th1 and Th2 polarization, T-bet and GATA-3 in naive ob/ob T cells. In this case, leptin was found to be necessary in T-helper 1- (Th1-) dependent inflammatory processes acting as a critical regulator of CD4+ T cell polarization in vitro and in vivo [66]. These data regarding leptin modulation of Th1-type cytokine production are in line with the observed effects of leptin stimulating TNF-*α* and IL-6 production by monocytes [24], further suggesting the possible role of human leptin in the regulation of the immune system inducing a proinflammatory response. On peripheral blood mononuclear cells of patients with ankylosing spondylitis, leptin exerts proinflammatory effect [67] as well as it enhances the proinflammatory cytokines in normal colonocytes and in HT29 xenografted tumor colonocytes. Colonocyte-derived products after leptin treatment stimulated perforin and granzyme B expressions in normal CD8 (+) T cells in vitro [68]. In addition, leptin alone or in combination with IL-1 enhanced the expression of iNOS and COX-2 and production of NO, PGE (2), IL-6, and IL-8. The effects of leptin are mediated through activation of transcription factor nuclear factor-kappaB (NF-kappaB) and mitogen-activated protein kinase (MAPK) pathway c-Jun NH (2)-terminal kinase (JNK).The increased synthesis of proinflammatory mediators is mediated by nitric oxide (NO) in human osteoarthritic cartilage [69].

On the other hand, leptin promotes T cell survival and Jurkat T lymphocytes survival [70] by modulating the expression of antiapoptotic proteins, such as Bcl-xL in stress-induced apoptosis [71]. This trophic effect of leptin on T cell is consistent with the reduction in lymphocyte numbers observed in fasted mice, that might be explained by the acute decrease in leptin levels [7, 19]. The role of leptin modulating T cell function in humans has been finally defined by clinical studies in specific and rare cases of patients with monogenic obesity. In human obesity due to congenital leptin deficiency, there is a T cell hyporesponsiveness (in addition to the expected neuroendocrine/metabolic dysfunction), and not only leptin treatment in these patients is an effective lowering body weight but it can also revert T cell response to mitogen activation in vitro [72]. In addition, leptin has been necessary in nonagenarians (≥90 years old) to maintain functional naive CD8 T cells and a healthy immune system [73].

The leptin receptor is highly expressed on the cell surface of Tregs. Leptin can act as a negative signal for the proliferation of human Foxp3 (+) CD4 (+) CD25 (+) regulatory T (T(reg)) cells. In vitro, neutralization with leptin monoclonal antibody (mAb), during anti-CD3 and anti-CD28 stimulation, resulted in T(reg) cell proliferation, which was interleukin-2 (IL-2) dependent. Together with the finding of enhanced proliferation of T(reg) cells observed in leptin- and ObR-deficient mice, these results suggest a potential for therapeutic interventions in immune and autoimmune diseases [74].

## 4. Conclusions

In conclusion, leptin plays a role in the activation of the immune system, and it is a mediator of inflammation. In this context, leptin may be one of the mediators responsible for the low-level systemic inflammation that may be present in metabolic syndrome-associated chronic pathologies such as atherosclerosis, which is associated with obesity, especially central obesity. Therefore, leptin may be considered as a therapeutic target in some clinical situations, such as proinflammatory states or autoimmune diseases, to control an excess of immune response, as well as in other clinical situations, such as starving, to control an excess of exercise, or immune deficiencies, to improve the impaired immune response. That is why the investigation of the role of leptin in the regulation of the immune response remains a challenge for the future.
